# Public attitudes toward COVID-19 vaccination: The role of vaccine attributes, incentives, and misinformation

**DOI:** 10.1038/s41541-021-00335-2

**Published:** 2021-05-14

**Authors:** Sarah Kreps, Nabarun Dasgupta, John S. Brownstein, Yulin Hswen, Douglas L. Kriner

**Affiliations:** 1 Department of Government, Cornell University, Ithaca, NY USA; 2 Injury Prevention Research Center, University of North Carolina, Chapel Hill, NC USA; 3 Department of Pediatrics, Harvard Medical School, Boston, MA USA; 4 Computational Epidemiology Lab, Boston Children’s Hospital, Boston, MA USA; 5 Epidemiology and Biostatistics, University of California, San Francisco, CA USA

**Keywords:** Translational research, Health care

## Abstract

While efficacious vaccines have been developed to inoculate against severe acute respiratory syndrome coronavirus 2 (SARS-CoV-2; also known as COVID-19), public vaccine hesitancy could still undermine efforts to combat the pandemic. Employing a survey of 1096 adult Americans recruited via the Lucid platform, we examined the relationships between vaccine attributes, proposed policy interventions such as financial incentives, and misinformation on public vaccination preferences. Higher degrees of vaccine efficacy significantly increased individuals’ willingness to receive a COVID-19 vaccine, while a high incidence of minor side effects, a co-pay, and Emergency Use Authorization to fast-track the vaccine decreased willingness. The vaccine manufacturer had no influence on public willingness to vaccinate. We also found no evidence that belief in misinformation about COVID-19 treatments was positively associated with vaccine hesitancy. The findings have implications for public health strategies intending to increase levels of community vaccination.

## Introduction

In less than a year, an array of vaccines was developed to bring an end to the SARS-CoV-2 pandemic. As impressive as the speed of development was the efficacy of vaccines such as Moderna and Pfizer, which are over 90%. Despite the growing availability and efficacy, however, vaccine hesitancy remains a potential impediment to widespread community uptake. While previous surveys indicate that overall levels of vaccine acceptance may be around 70% in the United States^1^, the case of Israel may offer a cautionary tale about self-reported preferences and vaccination in practice. Prospective studies^2^ of vaccine acceptance in Israel showed that about 75% of the Israeli population would vaccinate, but Israel’s initial vaccination surge stalled around 42%. The government, which then augmented its vaccination efforts with incentive programs, attributed unexpected resistance to online misinformation^3^.

Research on vaccine hesitancy in the context of viruses such as influenza and measles, mumps, and rubella, suggests that misinformation surrounding vaccines is prevalent^4,5^. Emerging research on COVID-19 vaccine preferences, however, points to vaccine attributes as dominant determinants of attitudes toward vaccination. Higher efficacy is associated with greater likelihood of vaccinating^6,7^, whereas an FDA Emergency Use Authorization^6^ or politicized approval timing^8^ is associated with more hesitancy. Whether COVID-19 misinformation contributes to vaccine preferences or whether these attributes or policy interventions such as incentives play a larger role has not been studied. Further, while previous research has focused on a set of attributes that was relevant at one particular point in time, the evidence and context about the available vaccines has continued to shift in ways that could shape public willingness to accept the vaccine. For example, governments, employers, and economists have begun to think about or even devise ways to incentivize monetarily COVID-19 vaccine uptake, but researchers have not yet studied whether paying people to receive the COVID-19 vaccine would actually affect likely behavior. As supply problems wane and hesitancy becomes a limiting factor, understanding whether financial incentives can overcome hesitancy becomes a crucial question for public health. Further, as new vaccines such as Johnson and Johnson are authorized, knowing whether the vaccine manufacturer name elicits or deters interest in individuals is also important, as are the corresponding efficacy rates of different vaccines and the extent to which those affect vaccine preferences. The purpose of this study is to examine how information about vaccine attributes such as efficacy rates, the incidence of side effects, the nature of the governmental approval process, identity of the manufacturers, and policy interventions, including economic incentives, affect intention to vaccinate, and to examine the association between belief in an important category of misinformation—false claims concerning COVID-19 treatments—and willingness to vaccinate.

## Results

### General characteristics of study population

Table 1 presents sample demographics, which largely reflect those of the US population as a whole. Of the 1335 US adults recruited for the study, a convenience sample of 1100 participants consented to begin the survey, and 1096 completed the full questionnaire. The sample was 51% female; 75% white; and had a median age of 43 with an interquartile range of 31–58. Comparisons of the sample demographics to those of other prominent social science surveys and U.S. Census figures are shown in Supplementary Table 1.

**Table 1:** Sample demographic.

	*N*	Percentage
Age		
18–29	240	(22%)
30–44	336	(31%)
45–59	277	(25%)
>= 60	243	(22%)
Sex		
Male	523	(48%)
Female	562	(51%)
Prefer not to say	11	(1%)
Race/ethnicity		
White	827	(75%)
Black	141	(13%)
Latinx	85	(8%)
Asian	70	(6%)
Native American	37	(3%)
Other	6	(1%)
Education		
Less than High School	25	(2%)
High School/GED	220	(20%)
Some College	308	(28%)
4-Year College Degree	224	(20%)
Graduate School	319	(29%)
Income		
< $20,000	197	(18%)
$20,000 to $39,999	205	(19%)
$40,000 to $59,999	166	(15%)
$60,000 to $79,999	133	(12%)
$80,000 to $99,999	100	(9%)
>= $100,000	295	(27%)
Political Partisanship		
Democrat (including those who leaned toward the party)	501	(46%)
Republican (including those who leaned toward the party)	513	(47%)
Political Ideology		
Very Liberal	166	(15%)
Liberal	161	(15%)
Somewhat Liberal	96	(9%)
Moderate	317	(29%)
Somewhat Conservative	89	(8%)
Conservative	106	(10%)
Very Conservative	161	(15%)

*Note:* Categories may not sum to 100% because of rounding.

### Vaccination preferences

Each subject was asked to evaluate a series of seven hypothetical vaccines. For each hypothetical vaccine, our conjoint experiment randomly assigned values of five different vaccine attributes—efficacy, the incidence of minor side effects, government approval process, manufacturer, and cost/financial inducement. Descriptions of each attribute and the specific levels used in the experiment are summarized in Table 2. After seeing the profile of each vaccine, the subject was asked whether she would choose to receive the vaccine described, or whether she would choose not to be vaccinated. Finally, subjects were asked to indicate how likely they would be to take the vaccine on a seven-point likert scale.

**Table 2:** Attributes and attribute levels for hypothetical COVID-19 vaccines.

Vaccine attributes	Levels
Efficacy	50%
	70%
	90%
Risk of mild side effects (fever, headaches, muscle pain)	1 in 2
	1 in 4
	1 in 10
Vaccine approval	Full FDA approval
	Emergency Use Authorization
Manufacturer	AstraZeneca
	Pfizer
	Moderna
	Johnson and Johnson
Cost or financial incentive	You pay: $20 co-pay
	Free
	You receive: $10 incentive
	You receive: $100 incentive

*Note:* Full text of the Full FDA approval and Emergency Use Authorization treatments are provided in the Supporting Information.

**Fig. 1:**
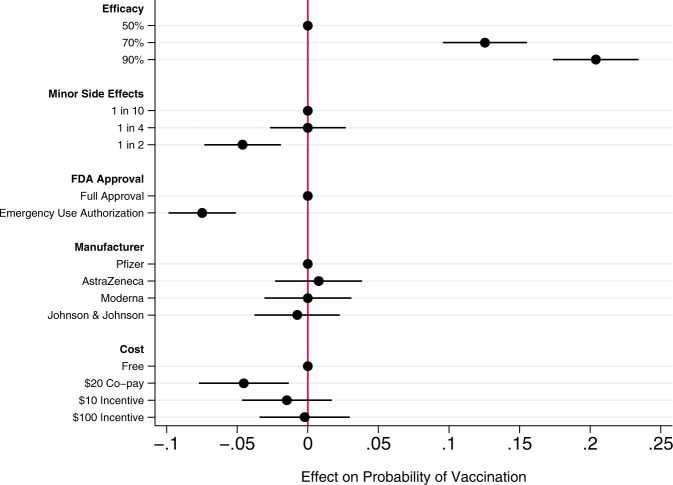
Effects of vaccine attributes on willingness to vaccinate. Circles present the estimated effect of each attribute level on the probability of a subject accepting vaccination from the attribute’s baseline level. Horizontal lines through points indicate 95% confidence intervals. Points without error bars denote the baseline value for each attribute. The average marginal component effects (AMCEs) are the regression coefficients reported in model 1 of Table 3.

Across all choice sets, in 4419 cases (58%) subjects said they would choose the vaccine described in the profile rather than not being vaccinated. As shown in Fig. 1, several characteristics of the vaccine significantly influenced willingness to vaccinate.

Efficacy had the largest effect on individual vaccine preferences. An efficacy rate of 90% increased uptake by about 20% relative to the baseline at 50% efficacy. Even a high incidence of minor side effects (1 in 2) had only a modest negative effect (about 5%) on willingness to vaccinate. Whether the vaccine went through full FDA approval or received an Emergency Use Authorization (EUA), an authority that allows the Food and Drug Administration mechanisms to accelerate the availability and use of treatments or medicines during medical emergencies^9^, significantly influenced willingness to vaccinate. An EUA decreased the likelihood of vaccination by 7% compared to a full FDA authorization; such a decline would translate into about 23 million Americans. While a $20 co-pay reduced the likelihood of vaccination relative to a no-cost baseline, financial incentives did not increase willingness to vaccinate. Lastly, the manufacturer had no effect on vaccination attitudes, despite the public pause of the AstraZeneca trial and prominence of Johnson & Johnson as a household name (our experiment was fielded before the pause in the administration of the Johnson & Johnson shot in the United States).

Model 2 of Table 3 presents an expanded model specification to investigate the association between misinformation and willingness to vaccinate. The primary additional independent variable of interest is a misinformation index that captures the extent to which each subject believes or rejects eight claims (five false; three true) about COVID-19 treatments. Additional analyses using alternate operationalizations of the misinformation index yield substantively similar results (Supplementary Table 4). This model also includes a number of demographic control variables, including indicators for political partisanship, gender, educational attainment, age, and race/ethnicity, all of which are also associated with belief in misinformation about the vaccine (Supplementary Table 2). Finally, the model also controls for subjects’ health insurance status, past experience vaccinating against seasonal influenza, attitudes toward the pharmaceutical industry, and beliefs about vaccine safety generally.

**Table 3:** Effects of vaccine attributes on willingness to vaccinate.

	(1)	(2)
Efficacy: 70%	0.13***	0.12***
	(0.02)	(0.01)
Efficacy: 90%	0.20***	0.20***
	(0.02)	(0.01)
Minor: 1 in 4	0.00	−0.00
	(0.01)	(0.01)
Minor: 1 in 2	−0.05***	−0.06***
	(0.01)	(0.01)
FDA: EUA	−0.07***	−0.08***
	(0.01)	(0.01)
Manufacturer: AstraZeneca	0.01	−0.01
	(0.02)	(0.01)
Manufacturer: Moderna	0.00	−0.01
	(0.02)	(0.01)
Manufacturer: Johnson & Johnson	−0.01	−0.01
	(0.02)	(0.01)
Cost: $20 Co-pay	−0.05***	−0.04***
	(0.02)	(0.01)
Cost: $10 Incentive	−0.01	−0.01
	(0.02)	(0.01)
Cost: $100 Incentive	−0.00	−0.00
	(0.02)	(0.01)
Vaccine safety beliefs		0.12***
		(0.01)
Misinformation index		0.01***
		(0.00)
Democrat		0.04
		(0.03)
Republican		0.00
		(0.03)
Female		−0.09***
		(0.02)
Age (in 10 years)		−0.02***
		(0.01)
Education		0.03***
		(0.01)
Past flu vaccination		0.02**
		(0.01)
Uninsured		0.02
		(0.02)
Pharma favorability		0.07***
		(0.01)
Black		−0.03
		(0.03)
Latinx		−0.04
		(0.04)
Constant	0.54***	−0.08
	(0.02)	(0.06)
Observations	7,672	7,357
R-squared	0.04	0.27

*Note*: Models are ordinary least squares regressions. Data presented as regression coefficients with robust standard errors clustered on respondent in parentheses. All significance tests are two-tailed.

*p *<* .10.

**p *<* .05.

***p *<* .01.

Greater levels of belief in misinformation about COVID-19 treatments were not associated with greater vaccine hesitancy. Instead, the relevant coefficient is positive and statistically significant, indicating that, all else being equal, individuals who scored higher on our index of misinformation about COVID-19 treatments were more willing to vaccinate than those who were less susceptible to believing false claims.

Strong beliefs that vaccines are safe generally was positively associated with willingness to accept a COVID-19 vaccine, as were past histories of frequent influenza vaccination and favorable attitudes toward the pharmaceutical industry. Women and older subjects were significantly less likely to report willingness to vaccinate than men and younger subjects, all else equal. Education was positively associated with willingness to vaccinate.

## Discussion

This research offers a comprehensive examination of attitudes toward COVID-19 vaccination, particularly the role of vaccine attributes, potential policy interventions, and misinformation. Several previous studies have analyzed the effects of vaccine characteristics on willingness to vaccinate, but the modal approach is to gauge willingness to accept a generic COVID-19 vaccine^10,11^. Large volumes of research show, however, that vaccine preferences hinge on specific vaccine attributes. Recent research considering the influence of attributes such as efficacy, side effects, and country of origin take a step toward understanding how properties affect individuals’ intentions to vaccinate^6–8,12,13^, but evidence about the attributes of actual vaccines, debates about how to promote vaccination within the population, and questions about the influence of misinformation have moved quickly^14^.

Our conjoint experiment therefore examined the influence of five vaccine attributes on vaccination willingness. The first category of attributes involved aspects of the vaccine itself. Since efficacy is one of the most common determinants of vaccine acceptance, we considered different levels of efficacy, 50%, 70%, and 90%, levels that are common in the literature^7,15^. Evidence from Phase III trials suggests that even the 90% efficacy level in our design, which is well above the 50% threshold from the FDA Guidance for minimal effectiveness for Emergency Use Authorization^16^, has been exceeded by both Pfizer’s and Moderna’s vaccines^17,18^. The 70% efficacy threshold is closer to the initial reports of the efficacy of the Johnson & Johnson vaccine, whose efficacy varied across regions^19^. Our analysis suggests that efficacy levels associated with recent mRNA vaccine trials increases public vaccine uptake by 20% over a baseline of a vaccine with 50% efficacy. A 70% efficacy rate increases public willingness to vaccinate by 13% over a baseline vaccine with 50% efficacy.

An additional set of epidemiological attributes consisted of the frequency of minor side effects. While severe side effects were plausible going into early clinical trials, evidence clearly suggests that minor side effects are more common, ranging from 10% to 100% of people vaccinated depending on the number of doses and the dose group (25–250 mcg)^20^. Since the 100 mcg dose was supported in Phase III trials^21^, we include the highest adverse event probability—approximating 60% as 1 in 2—and 1 in 10 as the lowest likelihood, approximating the number of people who experienced mild arthralgia^20^. Our findings suggest that a the prevalence of minor side effects associated with recent trials (i.e. a 1 in 2 chance), intention to vaccinate decreased by about 5% versus a 1 in 10 chance of minor side effects baseline. However, at a 25% rate of minor side effects, respondents did not indicate any lower likelihood of vaccination compared to the 10% baseline. Public communications about how to reduce well-known side effects, such as pain at the injection site, could contribute to improved acceptance of the vaccine, as it is unlikely that development of vaccine-related minor side effects will change.

We then considered the effect of EUA versus full FDA approval. The influenza H1N1 virus brought the process of EUA into public discourse^22^, and the COVID-19 virus has again raised the debate about whether and how to use EUA. Compared to recent studies also employing conjoint experimental designs that showed just a 3% decline in support conditional on EUA^6^, we found decreases in support of more than twice that with an EUA compared to full FDA approval. Statements made by the Trump administration promising an intensely rapid roll-out or isolated adverse events from vaccination in the UK may have exacerbated concerns about EUA versus full approval^8,23–25^. This negative effect is even greater among some subsets of the population. As shown in additional analyses reported in the Supplementary Information (Supplementary Fig. 5), the negative effects are greatest among those who believe vaccines are generally safe. Among those who believe vaccines generally are extremely safe, the EUA decreased willingness to vaccinate by 11%, all else equal. This suggests that outreach campaigns seeking to assure those troubled by the authorization process used for currently available vaccines should target their efforts on those who are generally predisposed to believe vaccines are safe.

Next, we compared receptiveness as a function of the manufacturer: Moderna, Pfizer, Johnson and Johnson, and AstraZeneca, all firms at advanced stages of vaccine development. Vaccine manufacturers in the US have not yet attempted to use trade names to differentiate their vaccines, instead relying on the association with manufacturer reputation. In other countries, vaccine brand names have been more intentionally publicized, such as Bharat Biotech’s *Covaxin* in India and Gamaleya Research Institute of Epidemiology and Microbiology *Sputnik V* in Russia. We found that manufacturer names had no impact on willingness to vaccinate. As with hepatitis and *H. influenzae* vaccines^26,27^, interchangeability has been an active topic of debate with coronavirus mRNA vaccines which require a second shot for full immunity. Our research suggests that at least as far as public receptiveness goes, interchangeability would not introduce concerns. We found no significant differences in vaccination uptake across any of the manufacturer treatments. Future research should investigate if a manufacturer preference develops as new evidence about efficacy and side effects becomes available, particularly depending on whether future booster shots, if needed, are deemed interchangeable with the initial vaccination.

Taking up the question of how cost and financial incentives shape behavior, we looked at paying and being paid to vaccinate. While existing research suggests that individuals are often willing to pay for vaccines^28,29^, some economists have proposed that the government pay individuals up to $1,000 to take the COVID-19 vaccine^30^. However, because a cost of $300 billion to vaccinate the population may be prohibitive, we posed a more modest $100 incentive. We also compared this with a $10 incentive, which previous studies suggest is sufficient for actions that do not require individuals to change behavior on a sustained basis^31^. While having to pay a $20 co-pay for the vaccine did deter individuals, the additional economic incentives had no positive effect although they did not discourage vaccination^32^. Consistent with past research^31,33^, further analysis shows that the negative effect of the $20 co-pay was concentrated among low-income earners (Supplementary Fig. 7). Financial incentives failed to increase vaccination willingness across income levels.

Our study also yields important insights into the relationship between one prominent category of COVID-19 misinformation and vaccination preferences. We find that susceptibility to misinformation about COVID-19 treatments—based on whether individuals can distinguish between factual and false information about efforts to combat COVID-19—is considerable. A quarter of subjects scored no higher on our misinformation index than random guessing or uniform abstention/unsure responses (for the full distribution, see Supplementary Fig. 2). However, subjects who scored higher on our misinformation index did not exhibit greater vaccination hesitancy. These subjects actually were more likely to believe in vaccine safety more generally and to accept a COVID-19 vaccine, all else being equal. These results run counter to recent findings of public opinion in France where greater conspiracy beliefs were negatively correlated with willingness to vaccinate against COVID-19^34^ and in Korea where greater misinformation exposure and belief were negatively correlated with taking preventative actions^35^. Nevertheless, the results are robust to alternate operationalizations of belief in misinformation (i.e., constructing the index only using false claims, or measuring misinformation beliefs as the number of false claims believed: see Supplementary Table 4).

We recommend further study to understand the observed positive relationship between beliefs in COVID-19 misinformation about fake treatments and willingness to receive the COVID-19 vaccine. To be clear, we do not posit a causal relationship between the two. Rather, we suspect that belief in misinformation may be correlated with an omitted factor related to concerns about contracting COVID-19. For example, those who believe COVID-19 misinformation may have a higher perception of risk of COVID-19, and therefore be more willing to take a vaccine, all else equal^36^. Additional analyses reported in the Supplementary Information (Supplementary Fig. 6) show that the negative effect of an EUA on willingness to vaccinate was concentrated among those who scored low on the misinformation index. An EUA had little effect on the vaccination preferences of subjects most susceptible to misinformation. This pattern is consistent with the possibility that these subjects were more concerned with the disease and therefore more likely to vaccinate, regardless of the process through which the vaccine was brought to market.

We also observe that skepticism toward vaccines in general does not correlate perfectly with skepticism toward the COVID-19 vaccine. Therefore, it is important not to conflate people who are wary of the COVID-19 vaccine and those who are anti-vaccination, as even medically informed individuals may be hesitant because of the speed at which the COVID-19 vaccine was developed. For example, older people are more likely to believe vaccines are safe but less willing to receive the COVID-19 vaccine in our survey, perhaps following the high rates of vaccine skepticism among medical staff expressing concerns regarding the safety of a rapidly-developed vaccine^2^. This inverse relationship between age and willingness to vaccinate is also surprising. Most opinion surveys find older adults are more likely to vaccinate than younger adults^37^. However, most of these survey questions ask about willingness to take a generic vaccine. Two prior studies, both recruiting subjects from the Lucid platform and employing conjoint experiments to examine the effects of vaccine attributes on public willingness to vaccinate, also find greater vaccine hesitancy among older Americans^6,7^. Future research could explore whether these divergent results are a product of the characteristics of the sample or of the methodological design in which subjects have much more information about the vaccines when indicating their vaccination preferences.

An important limitation of our study is that it necessarily offers a snapshot in time, specifically prior to both the election and vaccine roll-out. We recommend further study to understand more how vaccine perceptions evolve both in terms of the perceived political ownership of the vaccine—now that President Biden is in office—and as evidence has emerged from the millions of people who have been vaccinated. Similarly, researchers should consider analyzing vaccine preferences in the context of online vaccine controversies that have been framed in terms of patient autonomy and right to refuse^38,39^. Vaccination mandates may evoke feelings of powerlessness, which may be exacerbated by misinformation about the vaccines themselves. Further, researchers should more fully consider how individual attributes such as political ideology and race intersect with vaccine preferences. Our study registered increased vaccine hesitancy among Blacks, but did not find that skepticism was directly related to misinformation. Perceptions and realities of race-based maltreatment could also be moderating factors worth exploring in future analyses^40,41^.

Overall, we found that the most important factor influencing vaccine preferences is vaccine efficacy, consistent with a number of previous studies about attitudes toward a range of vaccines^6,42,43^. Other attributes offer potential cautionary flags and opportunities for public outreach. The prospect of a 50% likelihood of mild side effects, consistent with the evidence about current COVID-19 vaccines being employed, dampens likelihood of uptake. Public health officials should reinforce the relatively mild nature of the side effects—pain at the injection site and fatigue being the most common^44^—and especially the temporary nature of these effects to assuage public concerns. Additionally, in considering policy interventions, public health authorities should recognize that a $20 co-pay will likely discourage uptake while financial incentives are unlikely to have a significant positive effect. Lastly, belief in misinformation about COVID-19 does not appear to be a strong predictor of vaccine hesitancy; belief in misinformation and willingness to vaccinate were positively correlated in our data. Future research should explore the possibility that exposure to and belief in misinformation is correlated with other factors associated with vaccine preferences.

## Methods

### Survey sample and procedures

This study was approved by the Cornell Institutional Review Board for Human Participant Research (protocol ID 2004009569). We conducted the study on October 29–30, 2020, prior to vaccine approval, which means we captured sentiments prospectively rather than based on information emerging from an ongoing vaccination campaign. We recruited a sample of 1096 adult Americans via the Lucid platform, which uses quota sampling to produce samples matched to the demographics of the U.S. population on age, gender, ethnicity, and geographic region. Research has shown that experimental effects observed in Lucid samples largely mirror those found using probability-based samples^45^. Supplementary Table 1 presents the demographics of our sample and comparisons to both the U.S. Census American Community Survey and the demographics of prominent social science surveys.

After providing informed consent on the first screen of the online survey, participants turned to a choice-based conjoint experiment that varied five attributes of the COVID-19 vaccine. Conjoint analyses are often used in marketing to research how different aspects of a product or service affect consumer choice. We build on public health studies that have analyzed the influence of vaccine characteristics on uptake within the population^42,46^.

### Conjoint experiment

We first designed a choice-based conjoint experiment that allowed us to evaluate the relative influence of a range of vaccine attributes on respondents’ vaccine preferences. We examined five attributes summarized in Table 2. Past research has shown that the first two attributes, efficacy and the incidence of side effects, are significant drivers of public preferences on a range of vaccines^47–49^, including COVID-19^6,7,13,50^. In this study, we increased the expected incidence of minor side effects from previous research^6^ to reflect emerging evidence from Phase III trials. The third attribute, whether the vaccine received full FDA approval or an EUA, examines whether the speed of the approval process affects public vaccination preferences^6^. The fourth attribute, the manufacturer of the vaccine, allows us to examine whether the highly public pause in the AstraZeneca trial following an adverse event, and the significant differences in brand familiarity between smaller and less broadly known companies like Moderna and household name Johnson & Johnson affects public willingness to vaccinate. The fifth attribute examines the influence of a policy tool—offsetting the costs of vaccination or even incentivizing it financially—on public willingness to vaccinate.

Attribute levels and attribute order were randomly assigned across participants. A sample choice set is presented in Supplementary Fig. 1. After viewing each profile individually, subjects were asked: “If you had to choose, would you choose to get this vaccine, or would you choose not to be vaccinated?” Subjects then made a binary choice, responding either that they “would choose to get this vaccine” or that they “would choose not to be vaccinated.” This is the dependent variable for the regression analyses in Table 3. After making a binary choice to take the vaccine or not be vaccinated, we also asked subjects “how likely or unlikely would you be to get the vaccine described above?” Subjects indicated their vaccination preference on a seven-point scale ranging from “extremely likely” to “extremely unlikely.” Additional analyses using this ordinal dependent variable reported in Supplementary Table 3 yield substantively similar results to those presented in Table 3.

To determine the effect of each attribute-level on willingness to vaccinate, we followed Hainmueller, Hopkins, and Yamamoto and employed an ordinary least squares (OLS) regression with standard errors clustered on respondent to estimate the average marginal component effects (AMCEs) for each attribute^51^. The AMCE represents the average difference in a subject choosing a vaccine when comparing two different attribute values—for example, 50% efficacy vs. 90% efficacy—averaged across all possible combinations of the other vaccine attribute values. The AMCEs are nonparametrically identified under a modest set of assumptions, many of which (such as randomization of attribute levels) are guaranteed by design. Model 1 in Table 3 estimates the AMCEs for each attribute. These AMCEs are illustrated in Fig. 1.

### Analyzing additional correlates of vaccine acceptance

To explore the association between respondents’ embrace of misinformation about COVID-19 treatments and vaccination willingness, the survey included an additional question battery. To measure the extent of belief in COVID-19 misinformation, we constructed a list of both accurate and inaccurate headlines about the coronavirus. We focused on treatments, relying on the World Health Organization’s list of myths, such as “Hand dryers are effective in killing the new coronavirus” and true headlines such as “Avoiding shaking hands can help limit the spread of the new coronavirus^52^.” Complete wording for each claim is provided in Supplementary Appendix 1. Individuals read three true headlines and five myths, and then responded whether they believed each headline was true or false, or whether they were unsure. We coded responses to each headline so that an incorrect accuracy assessment yielded a 1; a correct accuracy assessment a -1; and a response of unsure was coded as 0. From this, we created an additive index of belief in misinformation that ranged from -8 to 8. The distribution of the misinformation index is presented in Supplementary Fig. 2. A possible limitation of this measure is that because the survey was conducted online, some individuals could have searched for the answers to the questions before responding. However, the median misinformation index score for subjects in the top quartile in terms of time spent taking the survey was identical to the median for all other respondents. This may suggest that systematic searching for correct answers is unlikely.

To ensure that any association observed between belief in misinformation and willingness to vaccinate is not an artifact of how we operationalized susceptibility to misinformation, we also constructed two alternate measures of belief in misinformation. These measures are described in detail in the Supplementary Information (see Supplementary Figs. 3 and 4). Additional regression analyses using these alternate measures of misinformation beliefs yield substantively similar results (see Supplementary Table 4). Additional analyses examining whether belief in misinformation moderates the effect of efficacy and an FDA EUA on vaccine acceptance are presented in Supplementary Fig. 6.

Finally, model 2 of Table 3 includes a range of additional control variables. Following past research, it includes a number of demographic variables, including indicator variables identifying subjects who identify as Democrats or Republicans; an indicator variable identifying females; a continuous variable measuring age (alternate analyses employing a categorical variable yield substantively similar results); an eight-point measure of educational attainment; and indicator variables identifying subjects who self-identify as Black or Latinx. Following previous research^6^, the model also controlled for three additional factors often associated with willingness to vaccinate: an indicator variable identifying whether each subject had health insurance; a variable measuring past frequency of influenza vaccination on a four-point scale ranging from “never” to “every year”; beliefs about the general safety of vaccines measured on a four-point scale ranging from “not at all safe” to “extremely safe”; and a measure of attitudes toward the pharmaceutical industry ranging from “very positive” to “very negative.”

### Reporting summary

Further information on research design is available in the Nature Research Reporting Summary linked to this article.

## Supplementary information

Supplementary Information

Reporting Summary

## Data Availability

All data and statistical code to reproduce the tables and figures in the manuscript and Supplementary Information are published at the Harvard Dataverse via this link: 10.7910/DVN/ZYU6CO.
