# Supplementary material for: Public attitudes toward COVID-19 vaccination: The role of vaccine attributes, incentives, and misinformation
Source: NPJ Vaccines. 2021 May 14;6:73. doi: 10.1038/s41541-021-00335-2 (PMC8121853; doi:10.1038/s41541-021-00335-2)
Supplement: Supplementary file 1 — Supplementary Information [file 41541_2021_335_MOESM1_ESM.pdf]

Supplementary Discussion

Comparative Sample Demographics

Supplementary Table 1 presents the demographics of our Lucid sample (n = 1,096) and compares its demographic composition to that of two other major social science surveys, the most recent versions of the American National Election Study and General Social Survey, as well as to demographics from the US Census American Community Survey.

Complete Wording of Misinformation Battery and FDA Approval and EUA treatments

Complete wording for the eight items in the misinformation battery (five false claims; three true statements) is provided in Supplementary Appendix 1.

Subjects who received the Full FDA approval treatment were told: "The vaccine has been approved and licensed by the US Food and Drug Administration. This certifies that the vaccine has been shown to be safe and effective in clinical trials."

Subjects who received the FDA EUA treatment were told: "The vaccine has received an emergency use authorization from the US Food and Drug Administration. This allows the expedited use of promising drugs that have not received full FDA approval, but that early results suggest are safe and may be effective in combatting the virus."

Sample Choice Set

All subjects were asked to evaluate seven hypothetical vaccine profiles. A sample choice set is presented in Supplementary Figure 1. The levels of each attribute for each vaccine were randomly assigned, and the order in which the attributes appeared in the choice set was randomized across survey respondents.

The Correlates of Belief in Misinformation Concerning Covid-19 Treatments

Aside from assessing the influence of the vaccine attributes randomly manipulated in the conjoint experiment on US adults' willingness to vaccinate, a secondary objective of the study was to assess the association between individual respondents' levels of belief in misinformation about COVID-19 treatments and willingness to vaccinate.

As described in the text, we asked subjects to evaluate the veracity of eight statements about COVID-19 treatments, five of which were false and three of which were true. Correct answers were scored a -1; incorrect answers were scored 1; and responses of unsure/don't know were scored 0. The resulting index of belief in misinformation ranged from – 8 to 8. The distribution of this index across our sample is presented in Supplementary Figure 2.

Our treatment knowledge question battery allowed us to construct two alternate measures of misinformation about COVID-19 treatments. The first is the simple additive count of false headlines about COVID-19 treatments that each subject believed. This distribution is shown in Supplementary Figure 3.

A second additive index measure again examines accuracy perceptions of the five false headlines, but assigns 1 point for each false headline believed, 0 points for an unsure response, and -1 points for a response that the headline is incorrect. The distribution of this measure is shown in Supplementary Figure 4.

Supplementary Table 2 presents a series of regression analyses modeling the factors associated with beliefs in COVID-19 misinformation. While there is some variation across models depending on the operationalization of belief in misinformation, these analyses broadly find that political partisanship, educational attainment, gender, and age are significantly associated with belief in misinformation.

Robustness Check: Alternate Operationalization of Willingness to Vaccinate

As described in the text, after seeing each hypothetical vaccine profile in the conjoint experiment, subjects were asked whether they would take the vaccine or choose not be vaccinated. This binary operationalization of willingness to vaccinate is the dependent variable for the analyses in the text.

After answering this binary question, we also asked subjects how likely they would be to take each vaccine. This variable is measured on a seven-point scale from extremely unlikely to extremely likely. As a robustness check, we re-estimated the analyses in Table 3 using this alternate operationalization of willingness to vaccinate. As shown in Supplementary Table 3, results are substantively similar.

Robustness Check: Modeling the Association between Willingness to Vaccinate and Alternate Operationalizations of Belief in Misinformation

As shown in Supplementary Table 4, re-estimating the regression analyses in Table 3 using the alternate measures of belief in misinformation described above yields substantively similar results. Most important, subjects who were more susceptible to believing COVID-19 misinformation were more likely to report willingness to vaccinate, all else being equal.

Moderating Role of Attitudes toward General Vaccine Safety on EUA Treatment

The EUA treatment significantly decreased willingness to vaccinate, on average, by 7%. However, additional analyses interacting the EUA treatment with the measure of general vaccine safety beliefs show that this effect is concentrated among those who believe vaccines are generally safe. As shown in Supplementary Figure 5, among those who believe vaccines generally are extremely safe, the estimated negative effect of the EUA treatment is 11%.

Moderating Role of Misinformation on EUA Treatment

Belief in misinformation also significantly moderated the effect of the EUA treatment. As shown in Supplementary Figure 6, the negative effect of the EUA is concentrated among subjects who scored lower on the COVID-19 treatment misinformation index. This is consistent with speculation that subjects who scored high on this index may be more concerned about COVID-19 in general, which in turn makes them more willing to vaccinate and less concerned by the EUA process vs. full FDA approval.

Moderating Role of Income on $20 Co-pay Treatment

Finally, Supplementary Figure 7 presents the results of another interaction model that examines how income moderates the negative effect of the $20 co-pay treatment on willingness to vaccinate. The dampening effect is large and statistically significant among low income subjects; as income increases, the effect wanes and becomes statistically insignificant.

Supplementary Figure 1: Sample Choice Set

As you may know, scientists around the world are working to develop a vaccine for Covid-19. Please consider the hypothetical vaccine described in the table below:

	**Vaccine 1**
**Efficacy – protection against severe symptoms such as respiratory failure, admission to a hospital ICU, or death**	70%
**Risk of mild side effects (flu-like symptoms)**	1 in 10
**Development and testing procedure**	The vaccine has received an emergency use authorization from the US Food and Drug Administration. This allows the expedited use of promising drugs that have not received full FDA approval, but that early results suggest are safe and may be effective in combatting the virus.
**Manufacturer**	Johnson & Johnson
**Cost or Financial Incentive**	Free

If you had to choose, would you choose to get this vaccine, or would you choose not to be vaccinated?

◯ I would choose to get this vaccine
◯ I would choose not to be vaccinated

Supplementary Figure 2: Histogram of Covid-19 Misinformation Index

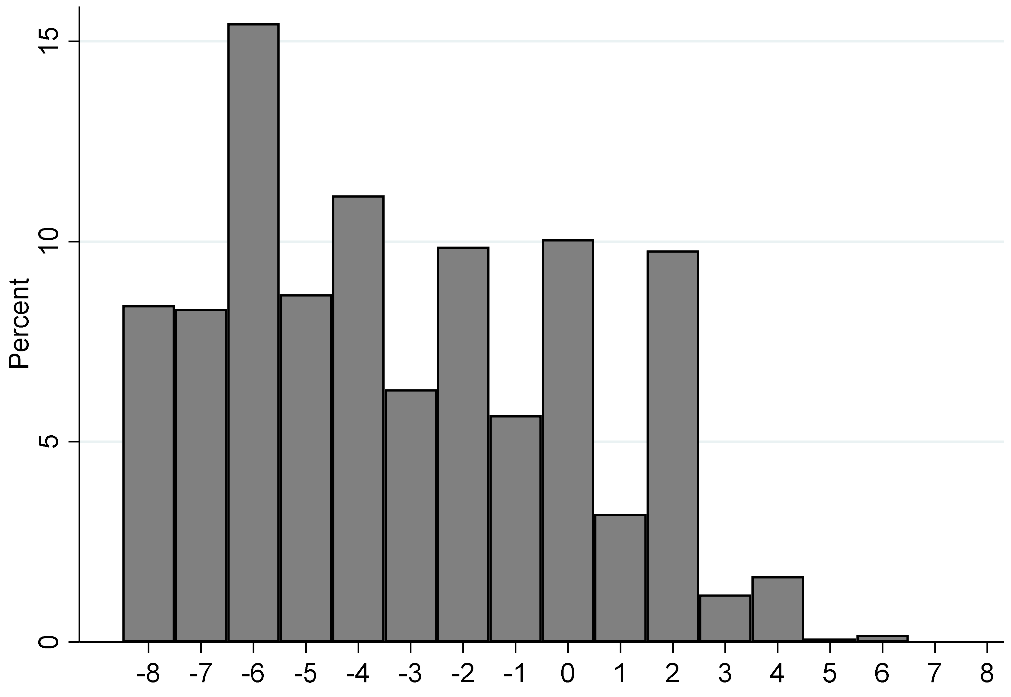

Supplementary Figure 3: Number of False Headlines Believed

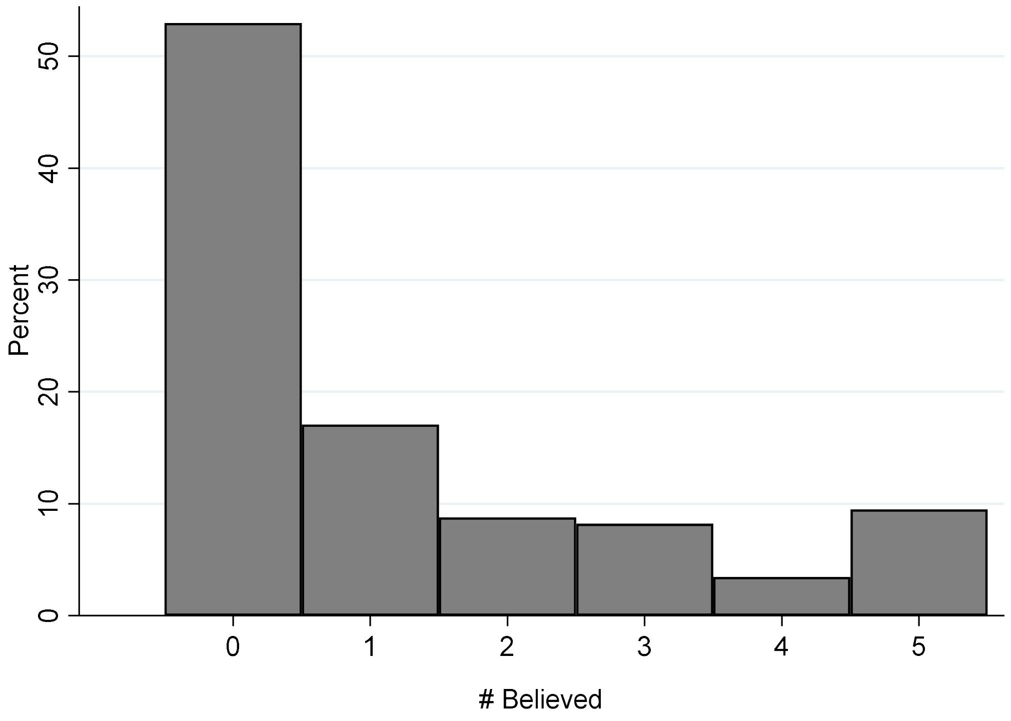

Supplementary Figure 4: Misinformation Index Using only False Headlines

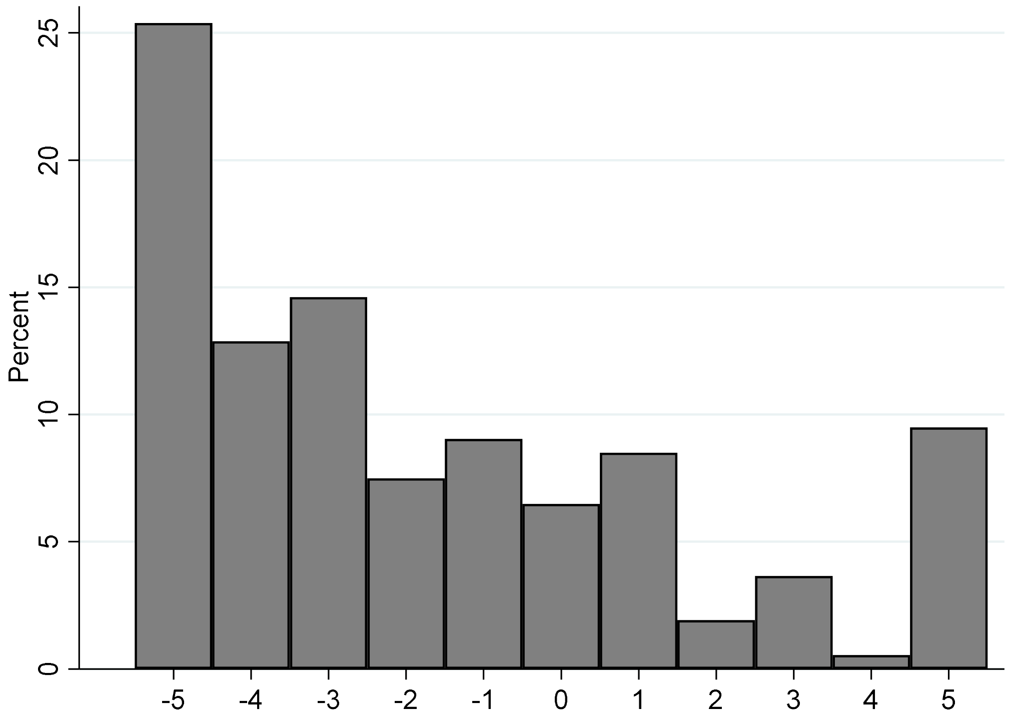

Supplementary Figure 5: General Vaccine Safety Attitudes Moderate EUA Effect

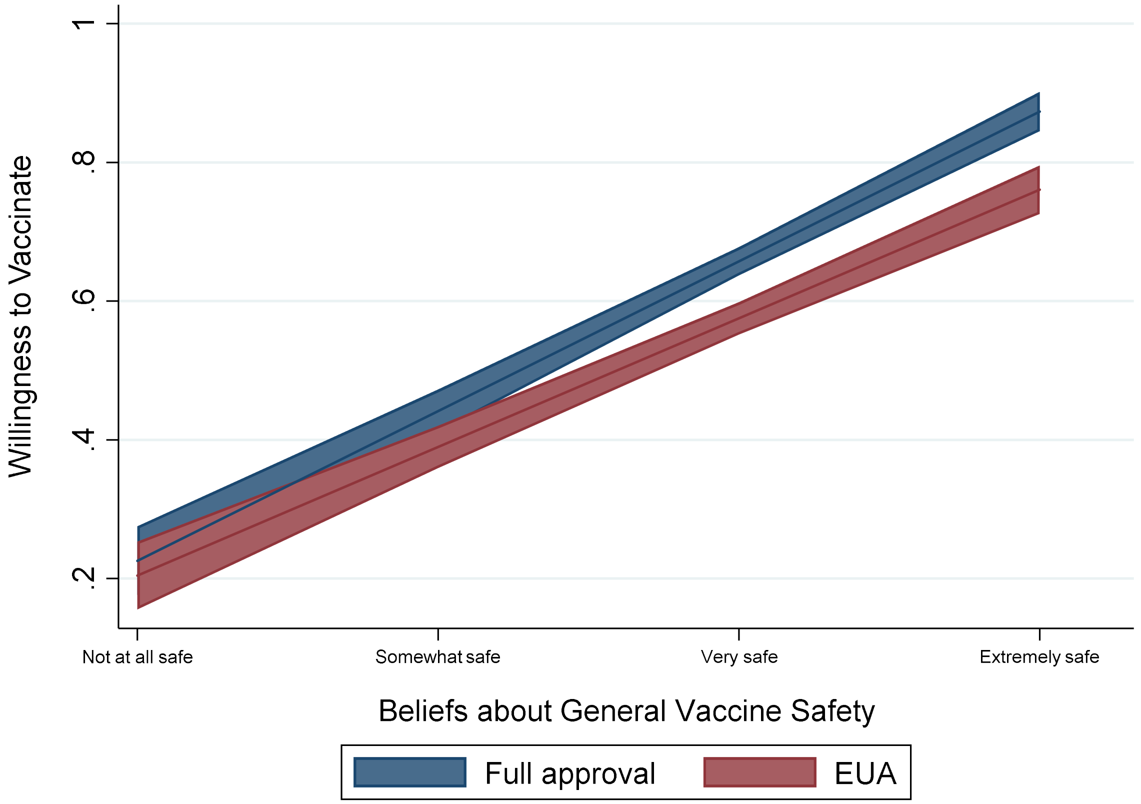

Supplementary Figure 6: Belief in Misinformation Moderates EUA Effect

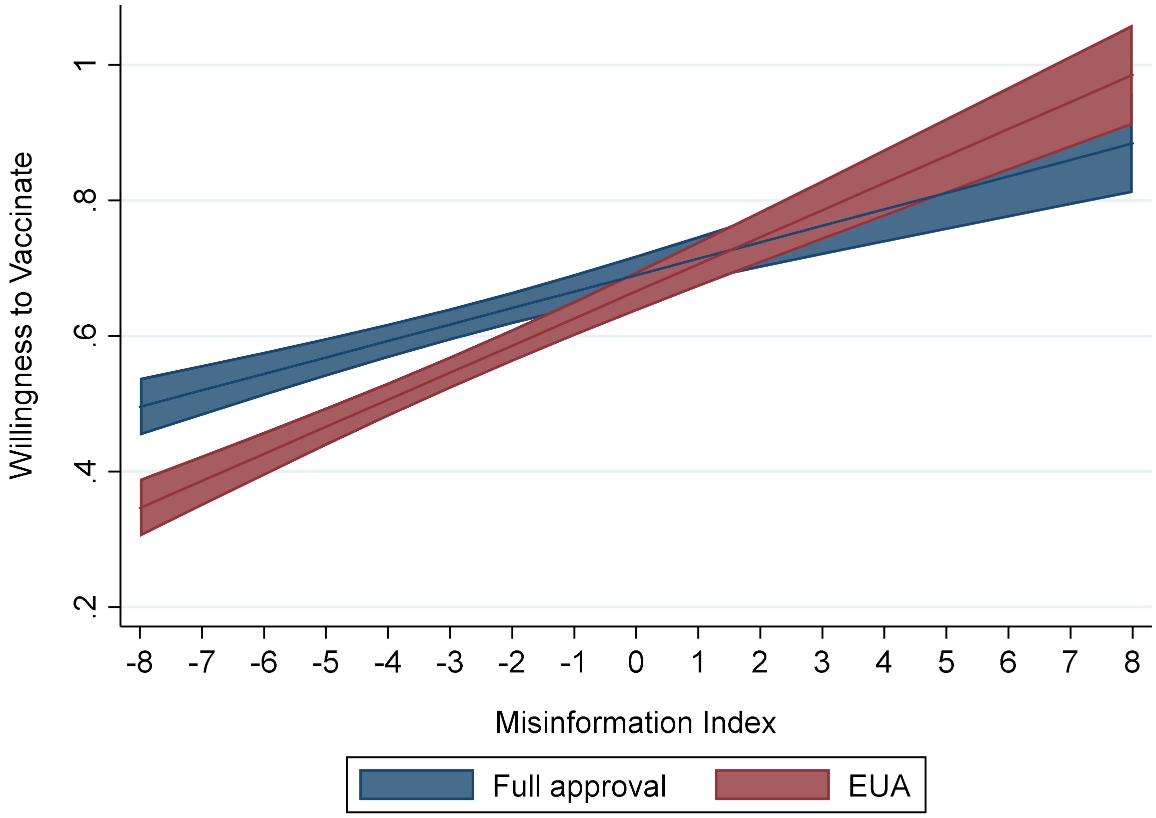

Supplementary Figure 7: Income Moderates Effect of Cost Treatment

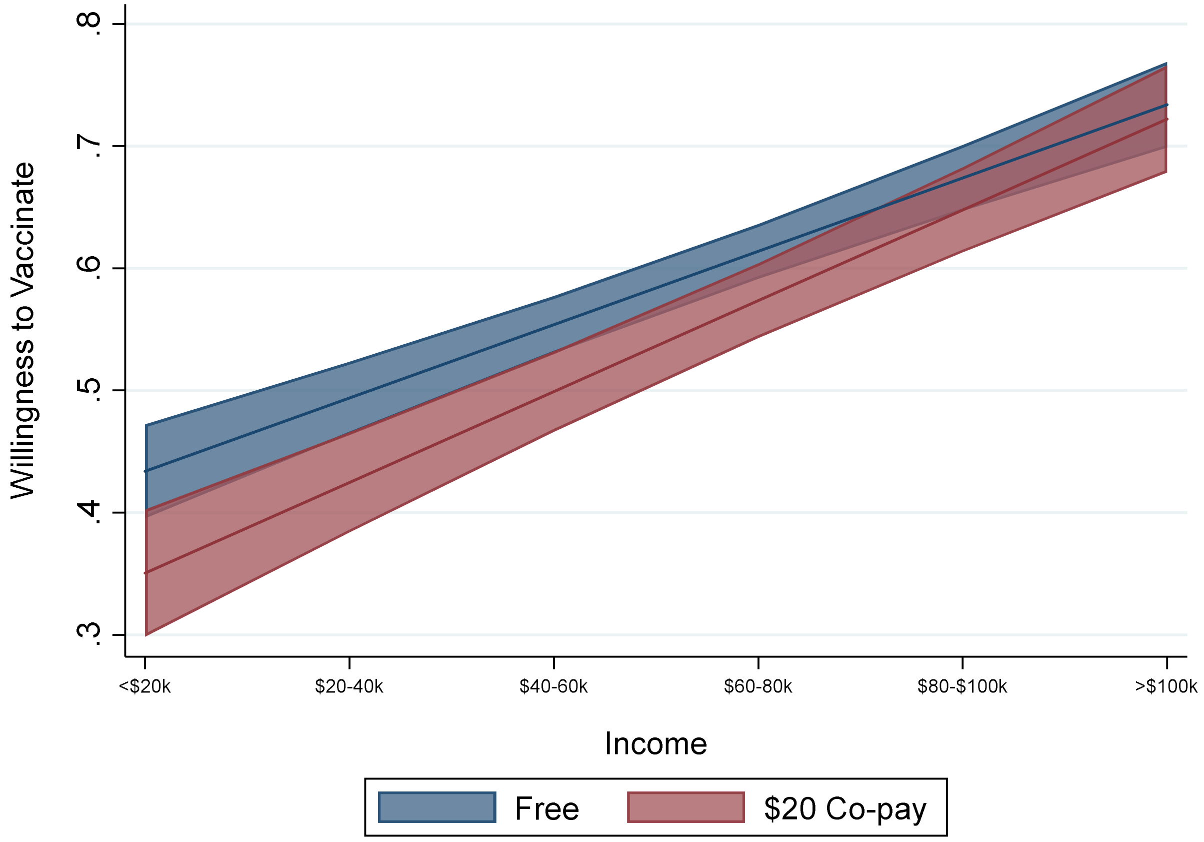

Supplementary Table 1: Comparative Demographics

	Lucid Sample	2016 ANES	2018 GSS	US Census
Demographics				
Black	13%	9%	16%	13%
Latino	8%	11%	6%	18%
Female	51%	52%	55%	51%
% College degree	51%	39%	33%	32%
Median age	42 years	49 years	48 years	38 years
Political Characteristics				
Republican	38%	29%	23%	
Democrat	38%	34%	32%	
Ideology (% moderates)	29%	21%	38%	

Note: All Census figures taken from the 2018 American Community Survey. Partisan measures do not include those who lean toward one party or the other.

Supplementary Table 2: Factors Predicting Extent of Belief in Misinformation

	(1)<br>All Items Index	(1)<br># False Believed	(3)<br>False Items Index
Democrat	-0.26	0.40***	0.15
	(0.24)	(0.12)	(0.24)
Republican	1.78***	0.95***	1.60***
	(0.24)	(0.12)	(0.24)
Education	0.08	0.13***	0.19***
	(0.05)	(0.02)	(0.05)
Female	-1.54***	-0.74***	-1.63***
	(0.18)	(0.09)	(0.17)
Age	-0.07***	-0.03***	-0.06***
	(0.01)	(0.00)	(0.01)
Black	0.53**	-0.10	0.05
	(0.27)	(0.13)	(0.26)
Latinx	-0.17	-0.10	-0.37
	(0.32)	(0.16)	(0.32)
Ln (alpha)		-0.38***	
		(0.13)	
Constant	-0.42	0.41*	0.01
	(0.40)	(0.21)	(0.40)
Observations	1,094	1,095	1,095
R-squared	0.29		0.26

Note: The first model is a negative binomial event count regression; models 2 and 3 are ordinary least squares regressions. Standard errors in parentheses. All significance tests are two-tailed.

* p < .10
** p < .05
*** p < .01

Supplementary Table 3: Effects of Vaccine Attributes on Willingness to Vaccinate (with Alternate Operationalization of Willingness to Vaccinate)

	(1)	(2)
Efficacy: 70%	0.52***	0.50***
	(0.07)	(0.06)
Efficacy: 90%	0.88***	0.85***
	(0.07)	(0.06)
Minor: 1 in 4	-0.02	-0.03
	(0.06)	(0.05)
Minor: 1 in 2	-0.16***	-0.21***
	(0.06)	(0.05)
FDA: EUA	-0.30***	-0.31***
	(0.05)	(0.05)
Manufacturer: AstraZeneca	0.00	-0.04
	(0.07)	(0.06)
Manufacturer: Moderna	-0.00	-0.04
	(0.07)	(0.05)
Manufacturer: Johnson & Johnson	-0.02	0.01
	(0.07)	(0.06)
Cost: $20 Co-pay	-0.15**	-0.14**
	(0.07)	(0.06)
Cost: $10 Incentive	-0.11	-0.11*
	(0.07)	(0.06)
Cost: $100 Incentive	-0.03	-0.05
	(0.07)	(0.06)
Vaccine safety beliefs		0.72***
		(0.06)
Misinformation index		0.07***
		(0.01)
Democrat		0.12
		(0.12)
Republican		0.03
		(0.13)
Female		-0.35***
		(0.09)
Age (in 10 years)		-0.01***
		(0.00)
Education		0.13***
		(0.02)
Past flu vaccination		0.10**
		(0.04)
Uninsured		0.13*
		(0.08)
Pharma favorability		0.43***

		(0.04)
Black		-0.15
		(0.13)
Latinx		-0.03
		(0.16)
Constant	4.38***	0.89***
	(0.10)	(0.28)
Observations	7,672	7,357
R-squared	0.03	0.39

Note: Models are ordinary least squares regressions. The dependent variable measures willingness to vaccinate on a seven-point scale from extremely unlikely to extremely likely. Robust standard errors clustered on respondent are in parentheses. All significance tests are two-tailed.

* $p < .10$
** $p < .05$
*** $p < .01$

Supplementary Table 4: Effects of Vaccine Attributes on Willingness to Vaccinate (with Alternate Operationalizations of Belief in Misinformation)

	(1)	(2)	(3)	(4)
Efficacy: 70%	0.13***	0.12***	0.12***	0.12***
	(0.02)	(0.01)	(0.01)	(0.01)
Efficacy: 90%	0.20***	0.20***	0.20***	0.20***
	(0.02)	(0.01)	(0.01)	(0.01)
Minor: 1 in 4	0.00	-0.00	-0.00	-0.00
	(0.01)	(0.01)	(0.01)	(0.01)
Minor: 1 in 2	-0.05***	-0.06***	-0.06***	-0.06***
	(0.01)	(0.01)	(0.01)	(0.01)
FDA: EUA	-0.07***	-0.08***	-0.08***	-0.08***
	(0.01)	(0.01)	(0.01)	(0.01)
Manufacturer: AstraZeneca	0.01	-0.01	-0.00	-0.01
	(0.02)	(0.01)	(0.01)	(0.01)
Manufacturer: Moderna	0.00	-0.01	-0.01	-0.01
	(0.02)	(0.01)	(0.01)	(0.01)
Manufacturer: Johnson & Johnson	-0.01	-0.01	-0.01	-0.01
	(0.02)	(0.01)	(0.01)	(0.01)
Cost: $20 Co-pay	-0.05***	-0.04***	-0.04***	-0.04***
	(0.02)	(0.01)	(0.01)	(0.01)
Cost: $10 Incentive	-0.01	-0.02	-0.02	-0.01
	(0.02)	(0.01)	(0.01)	(0.01)
Cost: $100 Incentive	-0.00	-0.00	-0.00	-0.00
	(0.02)	(0.01)	(0.01)	(0.01)
Vaccine safety beliefs		0.12***	0.12***	0.12***
		(0.01)	(0.01)	(0.01)
# False headlines believed		0.04***		
		(0.01)		
Misinformation items index			0.02***	
			(0.00)	
All items index				0.01***
				(0.00)
Democrat		0.03	0.04	0.04
		(0.03)	(0.03)	(0.03)
Republican		-0.01	0.00	0.00
		(0.03)	(0.03)	(0.03)
Female		-0.08***	-0.08***	-0.09***
		(0.02)	(0.02)	(0.02)
Age		-0.00***	-0.00***	-0.00***
		(0.00)	(0.00)	(0.00)
Education		0.02***	0.02***	0.03***
		(0.01)	(0.01)	(0.01)

Past flu vaccination		0.02***	0.02***	0.02**
		(0.01)	(0.01)	(0.01)
Uninsured		0.02	0.02	0.02
		(0.02)	(0.02)	(0.02)
Pharma favorability		0.07***	0.07***	0.07***
		(0.01)	(0.01)	(0.01)
Black		-0.02	-0.02	-0.03
		(0.03)	(0.03)	(0.03)
Latinx		-0.03	-0.03	-0.04
		(0.03)	(0.03)	(0.04)
Constant	0.54***	-0.12**	-0.07	-0.08
	(0.02)	(0.06)	(0.06)	(0.06)
Observations	7,672	7,364	7,364	7,357
R-squared	0.04	0.28	0.27	0.27

Note: Models are ordinary least squares regressions. Robust standard errors clustered on respondent are in parentheses. All significance tests are two-tailed.

* p < .10
** p < .05
*** p < .01

Supplementary Appendix 1: Misinformation Battery Questions

Subjects were asked to evaluate the accuracy of eight claims adapted from the World Health Organization's "Coronavirus disease (COVID-19) advice for the public: Mythbusters" webpage. Subjects evaluated each item as true, false, or could respond that they were unsure.

False Treatment Claims

- Spraying alcohol or chlorine all over your body can kill the new coronavirus.
- Hand dryers are effective in killing the new coronavirus.
- Antibiotics are effective in preventing and treating the new coronavirus.
- Cold weather and snow can kill the new coronavirus.
- Vaccines against pneumonia protect against the new coronavirus.

True Treatment Information

- Avoiding shaking hands can help limit the spread of the new coronavirus.
- Studies show hydroxychloroquine does not have clinical benefits in treating the new coronavirus.
- The FDA has authorized on an emergency basis the use of the Ebola drug, remdesivir, as well as convalescent plasma for the treatment of the new coronavirus.
